# Analytic method for evaluating players’ decisions in team sports: Applications to the soccer goalkeeper

**DOI:** 10.1371/journal.pone.0191431

**Published:** 2018-02-06

**Authors:** Leonardo Lamas, Rene Drezner, Guilherme Otranto, Junior Barrera

**Affiliations:** 1 Faculty of Physical Education, University of Brasilia, Brasilia, Federal District, Brazil; 2 School of Physical Education and Sport, University of São Paulo, São Paulo, São Paulo, Brazil; 3 Institute of Mathematics and Statistics, University of São Paulo, São Paulo, São Paulo, Brazil; Southwest University, CHINA

## Abstract

The aim of this study was to define a method for evaluating a player’s decisions during a game based on the success probability of his actions and for analyzing the player strategy inferred from game actions. There were developed formal definitions of i) the stochastic process of player decisions in game situations and ii) the inference process of player strategy based on his game decisions. The method was applied to the context of soccer goalkeepers. A model of goalkeeper positioning, with geometric parameters and solutions to optimize his position based on the ball position and trajectory, was developed. The model was tested with a sample of 65 professional goalkeepers (28.8 ± 4.1 years old) playing for their national teams in 2010 and 2014 World Cups. The goalkeeper’s decisions were compared to decisions from a large dataset of other goalkeepers, defining the probability of success in each game circumstance. There were assessed i) performance in a defined set of classes of game plays; ii) entropy of goalkeepers’ decisions; and iii) the effect of goalkeepers’ positioning updates on the outcome (save or goal). Goalkeepers’ decisions were similar to the ones with the lowest probability of goal on the dataset. Goalkeepers’ entropy varied between 24% and 71% of the maximum possible entropy. Positioning dynamics in the instants that preceded the shot indicated that, in goals and saves, goalkeepers optimized their position before the shot in 21.87% and 83.33% of the situations, respectively. These results validate a method to discriminate successful performance. In conclusion, this method enables a more precise assessment of a player’s decision-making ability by consulting a representative dataset of equivalent actions to define the probability of his success. Therefore, it supports the evaluation of the player’s decision separately from his technical skill execution, which overcomes the scientific challenge of discriminating the evaluation of a player’s decision performance from the action result.

## Introduction

In team sports, players’ decisions are determinants of the team’s performance. The several dynamic elements of the game (e.g., actions of team players and adversaries) and their stochastic nature require that players frequently make non-trivial decisions. The existence of a pre-conceived and well-designed player strategy, which provides a set of action alternatives for each context, and the ability of the player to choose the most adequate action from the strategy or from his personal repertory (i.e., player tactics) define the team’s success.

The control of any action execution has been modeled by a set of action rules of the following form: if <*condition*> then <*action*> [1]. According to this model, each player has a list of action rules available. They are strategically defined by the coaching staff, considering the actions a player should perform in each game circumstance. A player executes an action related to his list of action rules when his prediction of the next game state matches that action condition. Since an ill-conceived and well-executed action usually results in an unsuccessful outcome, it is important to analyze the decision that led to the action, not only its execution.

The variables underling the decision-making process have been investigated through distinct approaches, focusing on features of the perception-action process [2] and the functional interpersonal interactions between players [3–6]. Additionally, other studies have investigated the tactical efficiency of players’ decisions in adapted situations (i.e., small-sided games). For a review, see [7]. In this approach, evaluation instruments require that specialists judge the player’s choice. To evaluate a player’s decision through game observation and define a decision as not appropriate, it is necessary to propose an alternative decision and prove that it is better than the original one. However, if the player executes a different action, other players would act differently than in the original situation, and only trivial hypothetical contexts can be predicted.

In the literature, the problem of evaluating a player’s performance and of infering his most successful strategy based on alternative decisions has been approached by focusing on specific situations. For instance, the pick and roll in basketball [8], the penalty kick in soccer [9] or the service in tennis [10–11]. To evaluate a team’s performance, some studies have investigated the dynamics of collective actions in all game circumstances, without considering the players’ individual decisions [12–16]. In the present study, the problem of evaluating a player’s decisions will be approached by analyzing his efficiency in every game situation by estimating the success ratio of similar decisions made by other players in similar game situations.

The set of action rules of a player can be represented by a decision function that matches a game situation to an action. In a match, given intervenient factors (e.g., spatio-temporal pressure, level of stress, etc.), the player is not able to consistently make decisions in accordance with his action rules, characterizing his behavior as a stochastic process. The stochastic feature of a player’s performance will be considered to assess his decisions in a game through the following steps. First, a dynamic model of the set of situations analyzed in a sport is defined. Second, an experiment for sampling the dynamics of the considered game situations is designed. Third, an estimation based on sampled data of a stochastic process that models the action transitions of a player, given the evolvement of game situations, is made. Fourth, an estimation based on observed data and the stochastic model is made for a decision function of a player. Fifth, an estimation of a player’s decision function performance is made.

The described framework will be applied to analyze the specific context of the soccer goalkeepers’ decisions. It will be also presented a theoretically designed goalkeeper strategy. This strategy will be compared to strategies used by high-level goalkeeper in games analyzed in the experiment sample.

In the following sections, it is presented: i) a model of an individual player strategy in team sports and the stochastic properties of its application in a game; ii) an example of the player strategy in the context of the soccer goalkeeper; iii) the definition of the problem of a posteriori evaluation of a player’s decisions in sports and the methodological approach to solve it; iv) the experimental design for infering the goalkeepers’ strategy and for measuring their decision performance in games; v) the results of the analyzed goalkeepers’ decision performance; and vi) a discussion of the main results and conclusion of the work.

## Player strategy model

Team strategy is the control signal that orients each team’s players’ decisions in a game [1]. It has been modeled as a discrete dynamical system, whose states describe the specification of all team players’ actions and their concatenations. A player strategy (PS), in each team strategy, is a discrete dynamic system that specifies all actions of a player.

The PS can be represented by a connected direct graph. In this graph, a state is composed of three elements: i) state of movement input; ii) transformation; and iii) state of movement output. The state of movement is defined by the instantaneous dynamic of a player. The transformation is the execution of the action specified for that specific state. A state has two inputs: i) game situation and ii) metadata. Game situation is defined by the ball and all players’ dynamics. Metadata is defined by all information available previously to the game and its update during the game. Two states are connected based on the equivalence between the state of movement output of the first and the state of movement input of the second. A state can be connected to more than one state, and the continuation to one of them depends on which inputs satisfy the condition (i.e., game perception, metadata) of the action rule controlling the next state.

**Fig 1:**
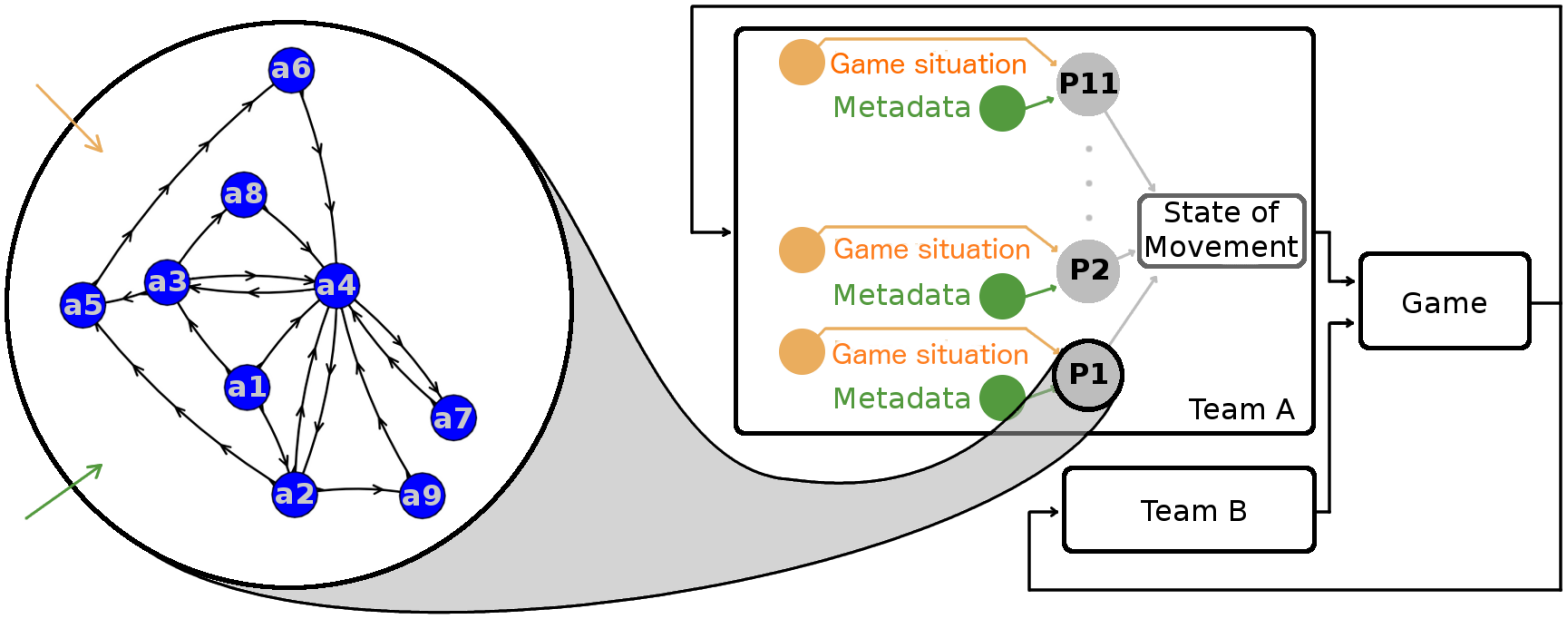
Graph of the goalkeeper’s possible actions. Part A: a_1_: start; a_2_: retrieved positioning, inside goalkeeper area; a_3_: advanced positioning, inside goalkeeper area; a_4_: linear displacement; a_5_: ready; a_6_: defense; a_7_: help defense; a_8_: frontal interception; a_9_: diagonal interception. Every node has an edge that connects directly to itself (not represented). Part B: gray nodes denote the eleven soccer team players (P_1_,…, P_11_); orange node: individual player game perception; green node: individual player set of metadata; P_1_,…, P_11_ decisions integrated in the state of movement. Both teams’ states of movement are inputs to the game process. Game feedback (black arrows departing from the game) is a source of the players’ subsequent decisions.

This dynamic system can be represented analytically by $a_{i+1}=\phi_{m_{i}}\left(a_{i},p_{i}\right)$, where *a*_*i*_ and *a*_*i*+1_ are, respectively, the current and the next state; *p*_*i*_ is the current game perception of the player; and *m*_*i*_ is the current metadata of the game.$\phi_{m_{i}}$ is called the decision function since it represents the choice of the next action. Given that the dynamics of the system are simply connected, $\phi_{m_{i}}$ must satisfy the following property: there exists a sequence of *n* inputs (*p*_*i*_, *m*_*i*_) such that *n* iterations of $\phi_{m_{i}}$ starting in an action *a* and activated by this sequence of inputs terminates in *a* itself. Fig 1—Part A presents an application of this model to describe the PS for a soccer goalkeeper.

The set of all PSs constitutes the team strategy (Fig 1—Part B). The collective orchestration of the team players (i.e., synchronization and coordination of actions) depends on the following individual elements: i) the perception of the game; ii) the metadata; and iii) the completeness of the set of action rules to support players’ interactions.

The representation of team strategy in terms of PSs (Fig 1—Part B) is equivalent to the representation presented by [1], since i) a team state can be represented in terms of individual players’ states; and ii) the PS has identical specifications to the one obtained by the concatenation of his actions in the team strategy. The execution of the PS in a game is not deterministic. Unpredicted game situations and spatio-temporal pressure are disturbing factors to the player’s decisions. Hence, a player’s decision (PD) can be defined as a stochastic process.

## Goalkeeper’s positioning model

This section presents a specific case of a player strategy model, the goalkeeper positioning model (GPM). For this purpose, positioning parameters and positioning rules based on the defined parameters were modeled.

### Goalkeeper’s positioning parameters

The soccer goalkeeper defensive roles can be divided in two categories: i) cover the defensive line and ii) defend the goal. First, cover the defensive line implies the protection of the space behind the closest defensive players to the goal. Second, defend the goal implies avoiding the ball reaching the goal. A goalkeeper uses a sequence of three classes of actions to defend the goal: positioning, ready pose and defense. Positioning displacement should direct the goalkeeper to a position that provides the greatest probability of making a defense in the moment of the shot, when he should be static in a ready pose, ready to perform a defense. In this process, the goalkeeper should make a sequence of decisions: i) position himself according to the ball and other players’ actions; ii) stop his displacement in the precise moment that precedes the shot; and iii) choose the appropriate defensive action for the type of shot.

**Fig 2:**
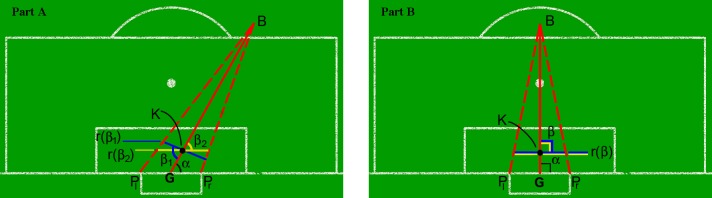
Soccer goalkeeper positioning parametric model. *B* is the ball position; *G* is the center of the goal; *P*_*l*_ and *P*_*r*_ are the left and right posts; *K* is the goalkeeper position on the pitch; *α* is the angle between $\left(\bar{GK}\right)$ and the goal line $\left(\bar{P_{l}P_{r}}\right)$; *β* is the angle between the ball line $\left(\bar{GB}\right)$ and the goalkeeper line; *r*(*β*_1_), in blue, and *r*(*β*_2_), in yellow, are, respectively, the goalkeeper position perpendicular to the ball line and parallel to the goal line.

Three parameters define the goalkeeper positioning (Fig 2 Parts A-B): i) the vertical component of the line segment $\left(\bar{GK}\right)$ between the center of the goal (*G*) and the goalkeeper’s position on the pitch (*K*); ii) the angle *α* between $\left(\bar{GK}\right)$ and the goal line, $\bar{P_{l}P_{r}}$, the line between the left (*P*_*l*_) and the right (*P*_*r*_) posts; and iii) the angle *β* between the goalkeeper line, defined by the goalkeeper’s right and left feet, and the ball line $\left(\bar{GB}\right)$. The goalkeeper line is denoted by *r*(*β*). When the goalkeeper stays on the ball line far (near) from the center of the goal, the covered lateral region increases (decreases), while the vertical region decreases (increases). Thus, in all game situations, the positioning parameters (i.e., vertical component of $\left(\bar{GK}\right)$, *α* and *β*) should be used to settle the goalkeeper position that maximizes his coverage of the goal. The goalkeeper line, *r*(*β*), has two extreme rotations (Fig 2—Part A): *r*(*β*_1_), perpendicular to the ball line and *r*(*β*_2_), parallel to the goal line. In *rβ*_1_, the goalkeeper position is always symmetric, while in *rβ*_2_, the position is symmetric just when the ball is centrally aligned to the penalty spot (Fig 2—Part B). Moreover, in *rβ*_1_, the goalkeeper is always facing the ball, while in *rβ*_2_, he is on this position just when the ball line coincides with the central line. Additionally, in *rβ*_1_, for all ball line angles, the perpendicular positioning to the ball line is the one that maximizes the goalkeeper’s lateral coverage.

The goalkeeper performs a sequence of decisions to continuously update the vertical component of $\bar{GK}$, *α* and *β*, while the ball is displaced, with the main objective of protecting the goal. Hence, depending on his prediction of a shot or a pass, he should choose an optimal position that anticipates his objective.

### Goalkeeper’s positioning rules

Herein, a set of goalkeeper positioning rules are modeled, with the dynamic attribution of values to the previously defined parameters (see Fig 2). The positioning rules encompass the goalkeeper’s displacements on the pitch for the different ball positions in each offensive situation. These rules intend to maximize the goal coverage area by the goalkeeper in all kinds of offensive situations.

**Fig 3:**
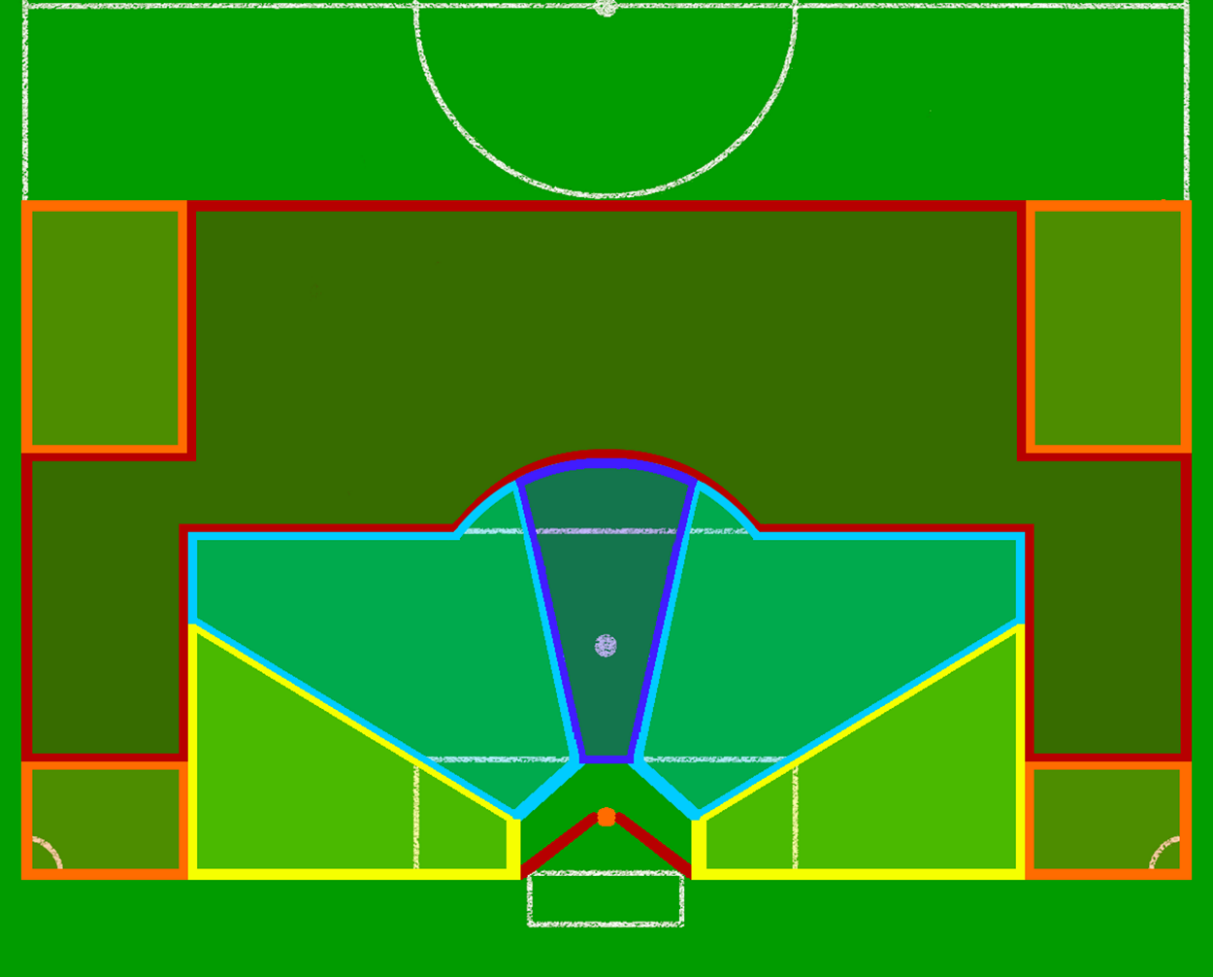
Model of goalkeeper positioning rules. Soccer pitch partitions indicate reciprocity between the goalkeeper positioning (colored lines in the two sets of polygonal lines in the goal area) and ball position (colored areas outside the goal area).

The pictorial representation of the positioning rules consists of partitioning the pitch into colored continuous regions (Fig 3). The goal area has been partitioned by two sets of colored connected polygonal lines. These partitions delimit the areas in which the goalkeeper should position for offensive situations with ground ball displacements. The goalkeeper should stay on the area of the same color that delimits the corresponding ball position. Particular situations are the transitions between regions. Colored lines assign the boundaries between regions in the pitch. Thus, when the ball is in one of these lines, the goalkeeper should position on his correspondent line.

The rules of dynamic attribution of values for the parameters of the goalkeeper positioning (vertical component of $\bar{GK}$, *α* and *β*, see Fig 2) consist of i) choosing the length of the vertical component of $\bar{GK}$ that defines an intersection between the ball line $\bar{GB}$ and the region between the polygonal lines inside the goal area, which limit the goalkeeper displacement paths; ii) updating *α* dynamically to keep $\bar{GK}$ on $\bar{GB}$ line while the ball moves; and iii) updating *β* to the angle of the closest polygonal line inside the goal area, which approximates the perpendicular position between the goalkeeper line *r*(*β*) and the ball line $\bar{GB}$.

When the ball is in one of the orange regions, the goalkeeper should be on the orange point in the center of the red polygonal line. In the case of an offensive transition from a red region to the orange region close to the end line, the goalkeeper has a specific displacement. He first follows the red polygonal line into the end line direction and then, when offense enters the orange area, he changes displacement direction until he reaches the orange point. When the ball is in the yellow, light or dark blue regions, the goalkeeper should be in the respective line.

## Evaluation of a player’s decisions

In team sports, a player’s decisions can be modeled as an optimization problem. For each class of game situations, the player chooses, from a set of possible actions, the one that may maximize his possibility of success. Other players perceive the action, and their reactions are influenced by the context transformation. Therefore, the success of a player’s decision depends on the subsequent adversary decisions. For instance, a soccer goalkeeper who decides to stay on the goal line to defend a shot executed from behind the penalty area may take the goal. After the play outcome has been verified, one may consider that he could have adopted a more advanced position to increase the chances of deflecting the ball. However, in this possible situation, he could have taken the goal after a parabolic shot. The limitation of evaluating a player’s decision is that the information extracted from the observation of a single play is insufficient for the analysis of the quality of the decision made by the player, given that there are other possible actions he could perform in that game situation and that each action would change the context due to the reaction of the other players. To compare the efficiency among the possible decisions for a game situation, it would be necessary to access the probability of success of the other possible actions. Thus, the problem of evaluating a player’s decisions has no solution from the isolated observation of a play.

To approach the problem of a player’s decision evaluation in a specific context (e.g., team sport, player role, competition level), it is necessary to assess the success probability of the player’s decisions in a game. Additionally, considering that the most recurrent decisions in specific game situations may correspond to the player’s strategy, the analysis of the success probability of these decisions allows us to evaluate his strategy performance. The success probability of every decision should be evaluated based on an *oracle*. An oracle is a probability distribution estimated from events contained in a dataset of games with similar competition level. It informs the success probability of a player’s decision in any game situation. Thus, the oracle can be applied to evaluate the performance of a given type of player action in a certain game situation. In this section, we present a model that can be applied to a generic team player and, afterwards, we specify a model of the soccer goalkeeper.

### Modeling the player’s decision

In a decision moment, the player observes the game situation and his present state (i.e., player position and action), predicts the next possible actions and chooses the one that he should perform. Therefore, the player’s decisions can be modeled by a decision function, which defines the next action. Empirically, the player’s decision cannot be directly assessed through human observation. Thus, the decision should be indirectly evaluated through by assessing the type of action performed. This fact leads to a simplified model of a player’s decisions, represented by a discrete Markovian process. In the player’s decision model (PD), the player’s action transition, from *a*_*i*_ to *a*_*i*+1_, depends on i) *x*_*i*_, a stochastic input defined by the ball dynamics and all other players’ dynamics (except by the evaluated player); and ii) *a*_*i*_, the evaluated player present action. Hence, the probability of transition from action *a*_*i*_ in the game situation *x*_*i*_ to the action *a*_*i*+1_ is

$${P(a}_{i+1}|{(x}_{i},a_{i}))$$ (1)

### Modeling the player strategy

A player strategy (PS) can be defined as a deterministic sub-graph of the player decision model. It is computed by a decision function *f* that maps the pair (*x*_*i*_, *a*_*i*_) to an action *a*_*i*+1_, which chooses the most likely action in a transition of the PD. Thus,

$$a_{i+1}=Arg(Max\{{P(a}_{j}|{(x}_{i},a_{i}),j\in J\})$$ (2)

is the set of all possible next actions for *a*_*i*_ in the situation *x*_*i*_.

### Modeling the oracle

The player’s decisions and the player’s strategy can be evaluated through comparison with an oracle defined for a given player profile (i.e., specific sport, role and competition level). The oracle is modeled by the join distribution

$$P(({(x}_{i},a_{i}){,a}_{i+1}),y)$$ (3)

of ((*x*_*i*_, *a*_*i*_), *a*_*i*+1_) and *y*. For any player of the considered profile, the pair ((*x*_*i*_, *a*_*i*_), *a*_*i*+1_) represents the next action *a*_*i*+1_ taken in accordance with the game situation *x*_*i*_ and the action *a*_*i*_, while the binary variable *y* represents success when true (i.e., *y* = 1) and fail (i.e., *y* = 0) otherwise. This join distribution should be estimated from a large sample of plays compatible with the competition level of the player profile.

### Modeling a soccer goalkeeper’s performance

A goalkeeper’s performance can be evaluated through the goal probability for each offensive situation. This evaluation criterion is herein defined as *absolute performance* (AP):

$$AP({(x}_{i},a_{i}){,a}_{i+1})(i+1)=P({(x}_{i},a_{i}){,a}_{i+1})(P(0|({(x}_{i},a_{i}){,a}_{i+1})))$$ (4)

Additionally, it is possible to evaluate a goalkeeper’s performance through the normalization of the goal probability by the goal potential of the considered situation. In a game situation *x*_*i*_, *the goal potential* is the probability of a goal given *x*_*i*_, that is, *P*(0|(*x*_*i*_). The *relative performance* (RP) in an instant *i* + 1 is associated with an action *a*_*i*+1_ that is selected from a situation *x*_*i*_, and the present action *a*_*i*_, is given by

$$RP({(x}_{i},a_{i}){,a}_{i+1})(i+1)=P({(x}_{i},a_{i}){,a}_{i+1})(P(0|({(x}_{i},a_{i}){,a}_{i+1}))-P{(0|x}_{i}))$$ (5)

The normalization of the RP by the goal potential allows the comparison of performance in different game situations. When RP < 0 and RP > 0 the analyzed goalkeeper has, respectively, a better and a worse performance than the goal potential for that situation.

## Experiment

In the previous section, a probabilistic model for evaluating a player’s decision was defined. In this experiment, the model is applied to analyze soccer goalkeeper’s decision performance regarding positioning. The evaluation encompasses both the goalkeeper’s decisions in a game (see PD, Eq 1) and the goalkeeper’s strategy (see PS, Eq 2) through the following steps: i) estimation of an oracle from the assessment of plays that resulted in goal or save from a sample of professional goalkeepers (oracle results have been used in the following steps (ii, iii and iv)); ii) evaluation of the goalkeeper’s decision performance from the annotation of game plays of two professional goalkeepers; iii) evaluation of the strategy of the two professional goalkeepers assessed; iv) evaluation of a goalkeeper’s positioning model (GPM, previously defined); and v) dynamic evaluation of a goalkeeper’s positioning in game plays.

### Experimental approach

The goalkeeper’s decision performance was evaluated during game plays. A game play was modeled as a categorical stochastic process, with the game situation (*x*_*i*_), the goalkeeper action (*a*_*i*_) and the respective outcome (*y*), for instance, the goal. The triple (*x*_*i*_, *a*_*i*_, *y*) defined a vector of labels that described the main events, which were sampled in systematic small time intervals of a game (every 2 Hertz). We considered only ball possessions that ended with a goalkeeper action.

**Fig 4:**
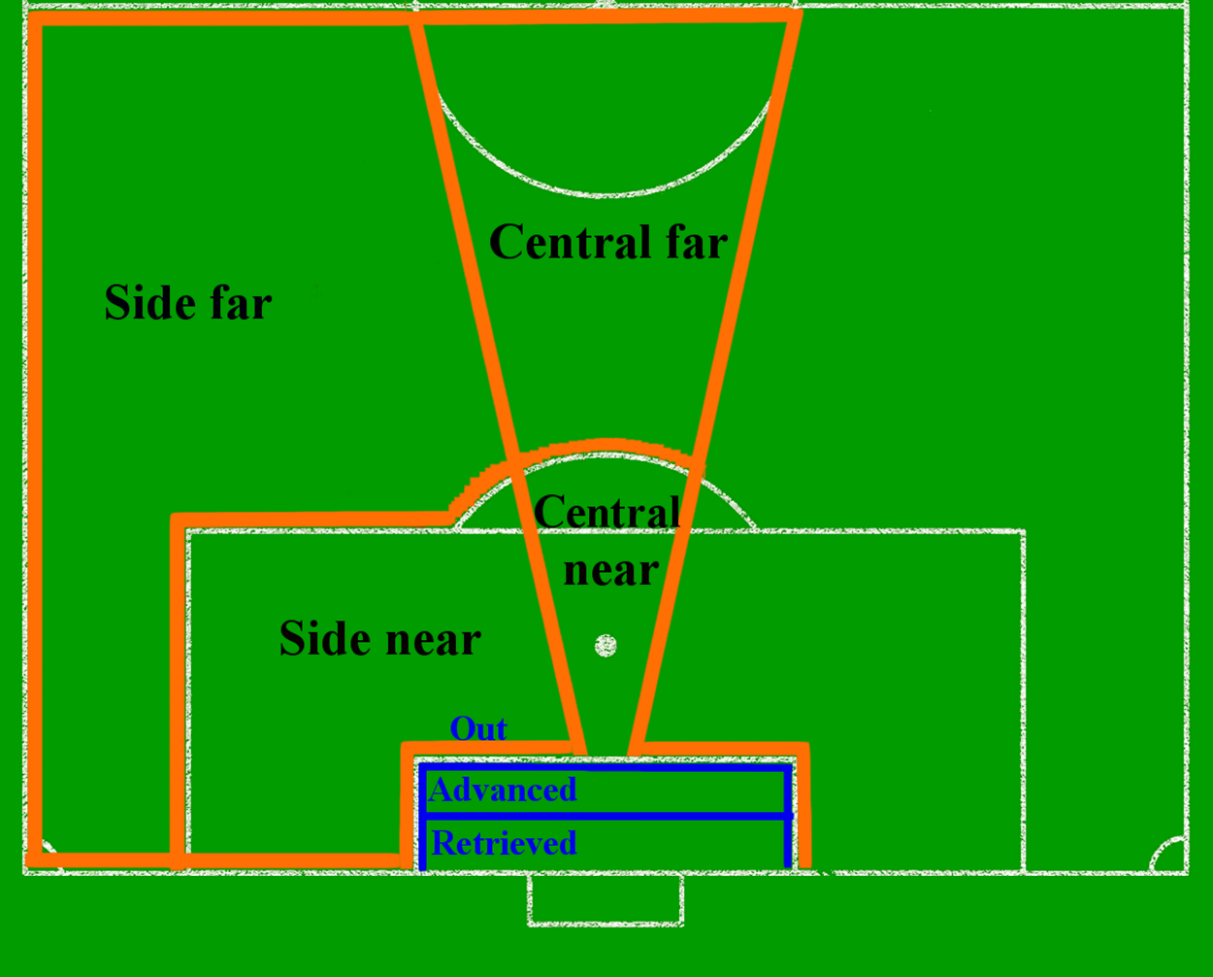
Classes of offensive situations. Orange delimited areas are as follows: i) side far; ii) central far; iii) side near; and iv) central near. Classes of goalkeeper positioning, blue delimited areas are as follows: i) retrieved; ii) advanced; and iii) out (of the goal area).

We defined the game situation (*x*_*i*_) by the following variables: i) ball player action; ii) ball zone; and iii) balance of the last defensive line. We modeled the ball player’s actions through the classes of ball circulation (i.e., passes to conserve ball possession, drives, dispute of the ball), cross and finalization. We modeled the ball zones through a partition of the pitch in 4 areas: i) central-near (i.e., a central area on the pitch and near the goal; ii) central-far; iii) side-near; and iv) side-far (Fig 4, orange delimited areas), which is a simplification of the pitch partitioning displayed in Fig 3. Side-near and side-far areas were considered equivalent in both the right and left sides of the pitch. Moreover, for the side-near and central-near zones, they were distinguished considering situations with and without cross. We modeled the balance/unbalance of the last defensive line considering whether or not there were defensive players, besides the goalkeeper, between the opponents and the goal area.

We defined the goalkeeper action (*a*_*i*_) by the following variables: i) goalkeeper positioning and ii) goalkeeper centralization. We modeled the goalkeeper position through 3 zones: i) retrieved, in the half of the goal area closest to the goal; ii) advanced, in the half of the goal area farthest to the goal; and iii) out of the goal area (Fig 4, blue areas). We modeled goalkeeper centralization by the combination of two variables, goalkeeper rotation and positioning relative to the ball line. The goalkeeper rotation considers the angle *β* between the goalkeeper line and the ball line (see Section Goalkeepers’ Positioning Parameters). The positioning relative to the ball line considers the alignment of the goalkeeper with the ball line. We have considered the goalkeeper centralized when he was rotated to the ball and aligned to the ball line. In contrary, the goalkeeper has been considered not centralized. We defined the game play outcome as goal or save.

Two types of analysis were performed, static and dynamic. In the static analysis, the goalkeeper’s decision performance regarding his positioning in the finalization moment was evaluated. For this purpose, we assessed the value of the pair (*x*_*i*_, *a*_*i*_) and compared it to the value of the equivalent pair in the oracle. There were considered the six classes of *x*_*i*_ for game plays with balanced defense (side-far; central-far; side-near cross; side-near not-cross; central-near cross; central-near not-cross). These classes were used for evaluating the positioning performance of the goalkeepers and the GPM.

In the dynamic analysis, we used the oracle data only. The samples of the two specific professional goalkeepers investigated were insufficient for this purpose. For all oracle data, we evaluated the effect of the sequence of positioning in the play outcome (goal or save) in the last three states before the finalization and the finalization state itself (1.5 seconds before the shot, with 2 Hertz sampling). Additionally, we selected two specific situations to illustrate the dynamic pattern of a game play: i) cross with finalization from central-near with balanced defensive; and ii) change in defensive balance resulting in finalization from side-near with unbalanced defense (i.e., situations that began with balanced defense and finished with unbalanced defense). In these two cases, we applied identical sampling procedures.

### Sample

We evaluated the decision performance of two professional goalkeepers, Gianluigi Buffon (GB) and Julio Cesar (JC), from the Italian and Brazilian national teams, respectively. GB was analyzed in the games of the UEFA Euro 2012 against Spain, Croatia, Republic of Ireland, England, Germany, and Spain, in a total of 77 plays with shots on goal. JC was analyzed in the games of the 2014 World Cup against Croatia, Mexico, Cameroon, Chile, Colombia, Germany, and the Netherlands, in a total of 57 plays with shots on goal.

To evaluate GB’s and JC’s performances, the oracle dataset was separated into two sub-sets: goals and saves. The goals sample comprised all the goals from the 2010 and 2014 World Cups, except for penalty kicks, direct free-kicks and deflected kicks. The saves sample contained a compilation of hard saves from the 2006, 2010 and 2014 World Cups. Hard saves encompassed all goalkeepers’ defenses with evident non-negligible positioning decisions demands, given the spatial and/or temporal pressure of the shot. The total oracle sample contained 399 game plays (284 goals and 115 saves). A single expert in soccer game analysis was responsible for the manual annotations of all game events. The Ethical Committee of the School of Physical Education and Sport of the University of São Paulo approved all experimental procedures (protocol: 2009–10).

### Reliability procedures

Reliability procedures were performed to assess the criteria consistency for the annotation of state variables (i.e., *x*_*i*_, *a*_*i*_, *outcome*), in accordance with the recommended standards for human annotations of sportive events [17]. Two raters with more than 10 years of experience in game analysis were asked to blindly analyze a set of 32 ball possessions with 192 sequences of positioning for each of four different professional goalkeepers. Reliability ratios were evaluated according to the levels of agreement for the Kappa value [18]: <0 less than chance agreement, 0.01–0.20 slight agreement, 0.21–0.40 fair agreement, 0.41–0.60 moderate agreement, 0.61–0.80 substantial agreement and 0.81–0.99 almost perfect agreement.

### Data analysis

Data analysis was based on the measures defined in section 4. In all analyses, the outcome *y* in the triple (*x*_*i*_, *a*_*i*_, *y*) was defined through a binary variable (goal, *y* = 0; save, *y* = 1). In the oracle sample, we estimated some variables: first, the *oracle average*, defined as the average goal probability of a situation *x*_*i*_, resulting from the outcomes of all events in this particular *x*_*i*_, *P*(*x*_*i*_|*y* = 0); second, the *oracle lowest*, defined as the best *a*_*i*_ performance, i.e., that with the lowest goal probability, for a specific pair (*x*_*i*_, *a*_*i*_). See Eq 3 for details. Events (*x*_*i*_, *a*_*i*_) with fewer than 5 occurrences were not considered in the oracle.

To evaluate a goalkeeper’s PS, Eq 2 was applied to define the most frequent *a*_*i*+1_ for a given context (*x*_*i*_, *a*_*i*_). Additionally, for a given *x*_*i*_, to evaluate the PD performance of a specific goalkeeper, we calculated the $\sum_{a_{i}}^{n}P(a_{i},x_{i})(P(a_{i}|y=0)),$ and we obtained the value attributed to *y* from the oracle. Entropy measure was applied to analyze the consistency of a goalkeeper’s PD performance regarding the specifications of his PS. In this sense, entropy indicates the variability of a goalkeeper’s actions in response to a specific game situation. High entropy indicates great variability in the goalkeeper’s actions. The entropy of a random discrete variable X with a probability distribution *P*, *with P*(*i*) = *p*_*i*_, was defined by [19] as follows:

$$H\left(X\right)=-\sum_{i=0}^{n-1}\;p_{i}log\;p_{i}.$$

To compare the goalkeeper entropy between game situations, we normalized the results to the maximum possible entropy considering the parameters of analysis (six possible different responses, with a maximum value of 2.58).

Finally, in the discrete analysis of the goalkeepers’ performance, both PD and PS were applied to GB’s and JC’s performances. Additionally, we analyzed GPM performance through comparison with the oracle data.

In the dynamic analysis, all data points in the sampling before the finalization state were matched to the oracle data corresponding to the goalkeeper position in finalization states. The oracle data were grouped in quartiles of goal probability (≤ 0.25; > 0.25 and ≤ 0.50; > 0.50 and ≤ 0.75; > 0.75). In the sequences analyzed, we assessed the variation of goal probability along the four data points and defined four shape profiles: constant, downward, upward and oscillating. For an oscillating shape, the last variation defined the trend of the curve (increment or decrement of the goal probability). We compared curve shapes between saves and goals.

## Results

### Intra- and inter-rater reliability test

The results of the intra- and inter-rater reliability tests for the criteria of the goalkeeper’s performance parameters are displayed in Table 1. The Kappa level of agreement was greater than 0.80 indicating an almost perfect intra- and inter-rater agreement [18].

**Table 1:** Intra- and inter-rater reliability for the criteria of a goalkeeper’s performance parameters (data expressed in percentage).

	Observer 1	Observer 2	Observer 1 x Observer 2
**Ball area**	0.88	0.91	0.80
**Goalkeeper positioning**	0.94	0.95	0.82
**Goalkeeper centralization**	1	0.89	0.93
**Balance of last defensive line**	0.99	0.98	0.95

### Performance of professional goalkeepers and GPM

The goal potential results for the oracle, for the PD and for the PS of professional goalkeepers and for the GPM, in balanced defense situations, are displayed in Table 2. Oracle (average) presented a moderate to high (0.59–0.85) and low to moderate (0.40–0.63) goal potentials, for “near” and “far” situations, respectively. The professional goalkeepers GB and JC performed consistently better than the oracle (average) and similarly to the oracle (lowest) in all offensive situations. Both goalkeepers evaluated presented better performance for PS than PD. Finally, the GPM performed equivalently to the best goalkeepers and the oracle results for all situations.

**Table 2:** Values (in percentage) of goal potential for the oracle (average and lowest values), the professional goalkeepers GB and JC and the GPM.

	Central Far	Side Far	Central Near (cross)	Central Near (not cross)	Side Near (cross)	Side Near (not cross)
Oracle (average)	0.63	0.40	0.85	0.82	0.69	0.59
Oracle (lowest)	0.50 RC	0.08 RC	0.57 RC	0.20 AC	0.42 RC	0.25 AC
GB (PD)	0.59	0.23	0.69	0.2(2 events)	0.45	0.26
GB (PS)	0.50 RC	0.20 AC	0.57 RC	0.20 AC	0.42 RC	0.25 AC
JC (PD)	0.56	0.18	0.70	1(1 event)	0.58	0.37
JC (PS)	0.50 RC	0.08 RC	0.57 RC	1(1event) ANC	0.42 RC	0.25 AC
GPM	0.50 RC	0.08 RC	null	0.20 AC	Null	0.25 AC

Goalkeeper positioning: i) retrieved inside the goal area and centralized (RC); ii) advanced inside the goal area and centralized (AC); iii) advanced inside the goal area and not centralized (ANC). PD: player decision model; PS: player strategy. GPM: geometrical positioning model.

**Fig 5:**
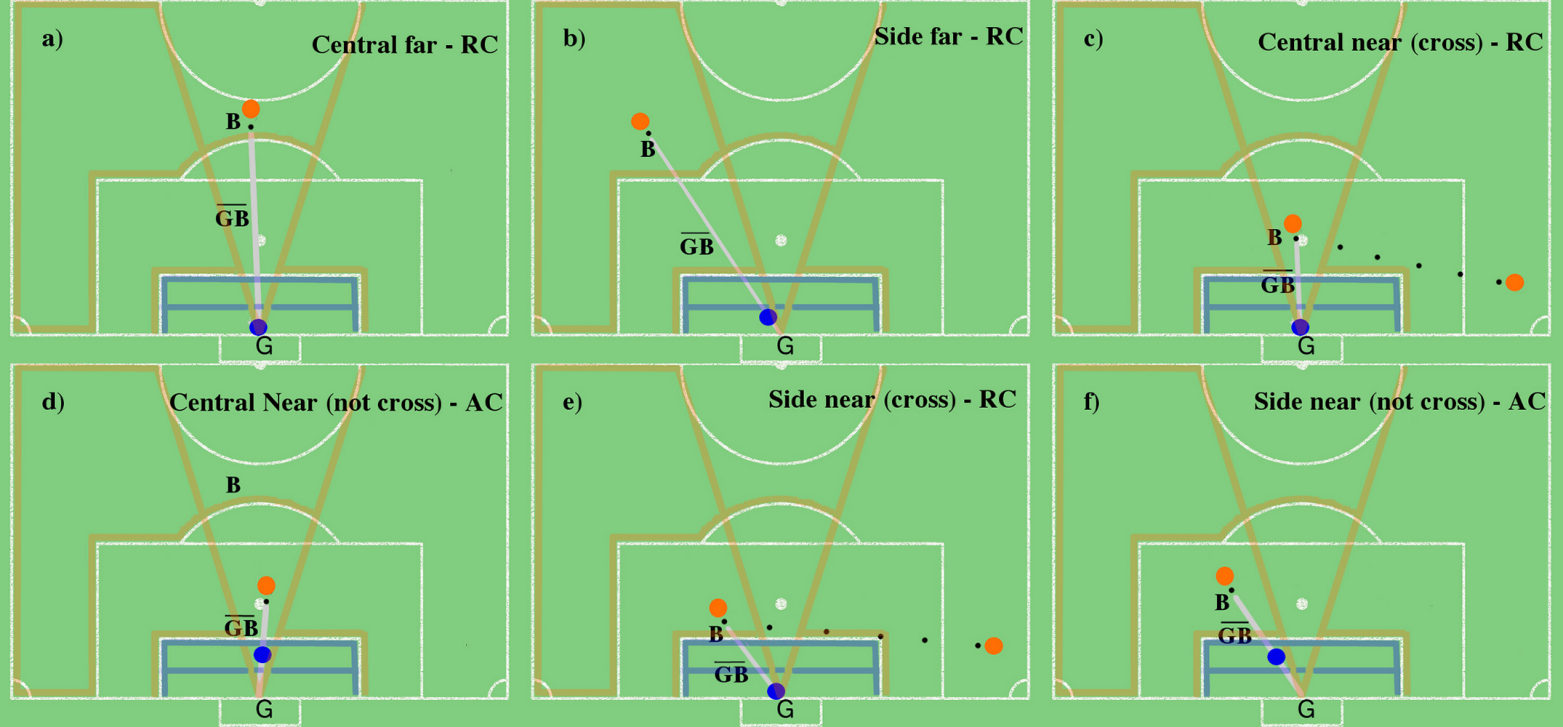
Representation of the pair (*x*_*i*_, *a*_*i*_) for each situation of the oracle lowest. Orange and blue circles are attackers and goalkeeper, respectively; the black circle is the ball; and $\bar{GB}$ is the ball line.

To clarify the positioning model adopted, Fig 5 illustrates the situations (*x*_*i*_, *a*_*i*_) and the corresponding statistics are presented in Table 2. The situations presented in Fig 5a–5f are those with balanced defenses and the best goalkeeper performance (oracle lowest in Table 2).

Alternatively, for unbalanced last defensive line situations, the oracle average of goal potential in the central-near situation was 0.91, and the oracle lowest was 0.67 equally for RC and AC. Additionally, the oracle average and lowest results of goal potential in the side-near were 0.72 and 0.29 for out of goal area and centralized (OC), respectively. Other types of positioning did not have occurrences in unbalanced situations. Both for the professional goalkeepers and the theoretical model of positioning, the unbalanced last defensive line situations were not considered because of their great diversity.

### Professional goalkeepers’ entropy

The professional goalkeepers’ entropy for each of the offensive situations are displayed in Table 3. The entropy remained low to moderate in most of the situations for both goalkeepers, suggesting they made consistent positioning choices. Complementarily, a few situations presented high entropy and were different between goalkeepers. For GB and JC, the highest entropies were achieved, respectively, in central-far (71%) and side-near not cross (68%).

**Table 3:** Professional goalkeepers’ entropies for each of the offensive situations. Absolute and (percentage) of the maximum entropy (2.58) for each offensive situation.

	Central Far	Side Far	Central Near (cross)	Central Near (not cross)	Side Near (cross)	Side Near (not cross)
GB	1.83 (0.71)	1.34 (0.52)	1.16 (0.45)	null	0.82 (0.32)	0.62 (0.24)
JC	1.25 (0.48)	1.36 (0.53)	1.25 (0.48)	Null	0.99 (0.38)	1.75 (0.68)

### Performance tendencies in goal and save situations

In the temporal analysis of goal and save situations, 34 events from the oracle sample were discarded because at least one data point of the sequence previous to the shoot had no oracle data to compare with. Total sample was composed of 365 events, with 263 goals and 102 hard saves. The temporal trends of goal potential in goal and save situations indicated particular patterns for each outcome. In goal situations, there was a high occurrence of upward (48.7%) and constant (33.1%) shapes in opposition to downward shapes (18.2%). In contrast, save situations presented a high occurrence of constant (42.1%) and downward shapes (41.2%) in opposition to upward shape (16.7%). In goals and saves, goal potential tended to, respectively, increase and decrease before the shoot.

**Fig 6:**
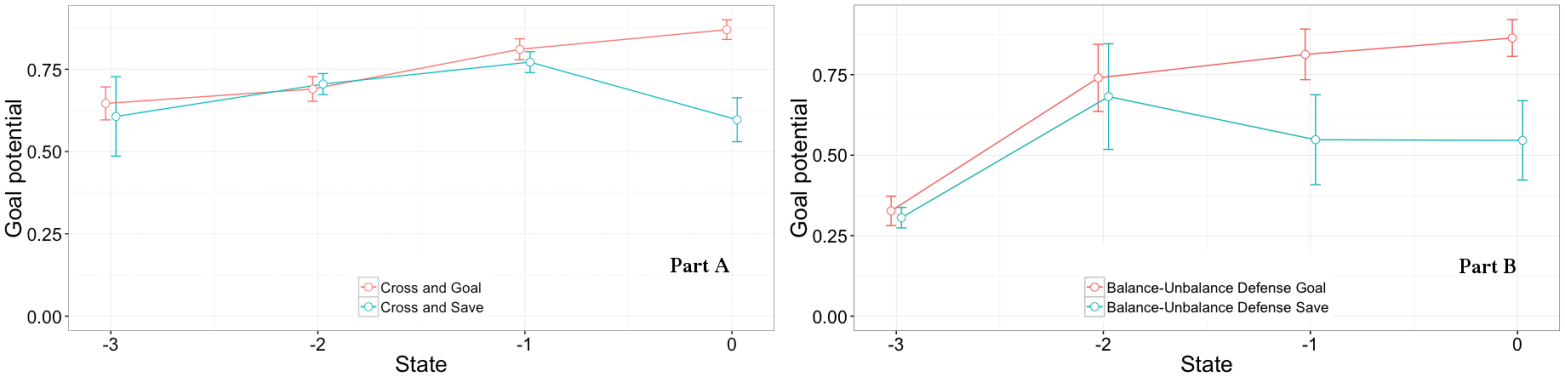
Goal potential trends. Part A: Cross situation (goal or save); Part B: Balanced/unbalanced last defensive line situation (goal or save), along the three states before the finalization and the finalization state.

To present the idea of the dynamic approach of goalkeepers’ performance, two exemplar situations were selected: crosses following a specific path (side-far, side-near, central-near) and balanced-unbalanced defenses. For crosses (Fig 6A), the difference between saves and goals occurred only in the last state (finalization state), with a prominent upward shift of the curve indicating increment of goal potential in goal situations. The main observed difference between goals and saves was related to the centralization variable. In goals and defense situations, the goalkeeper centralized his position in the last state in 21.87% and 83.33% of the episodes, respectively. For balanced-unbalanced last defensive line (with finalization in side-near), Fig 6B, the difference between goals and saves appeared in the state before the finalization. In goals and saves situations, the goalkeeper centralized his position in the finalization state 10% and 60% of the time, respectively.

## Discussion

The main contribution of the present study was to define an analytical method for evaluating the decision performance of a player. Formal definitions for i) the stochastic process of the player decision in game situations and ii) the inference process of the player strategy based on his game decisions were developed. In the specific context of soccer goalkeepers, a positioning model, with geometric parameters and solutions to optimize goalkeeper position based on the ball position and trajectory, was developed. Model parameters were used to create a dataset of similar situations performed by players of the identical role and the same competitive level (the oracle). Then, with the oracle defined, its probability distribution of successful and unsuccessful results could be used to grade the decisions of any analyzed player. This probabilistic approach overcomes the problem of discriminating the evaluation of a player’s decision performance from the action result.

At the moment, the framework has been applied to the evaluation of top professional goalkeepers. Experimental results indicated decisions were similar to the lowest probabilities of goal in the oracle, although the goalkeepers could improve their positioning consistency, as suggested by the entropy results. Additionally, positioning dynamics in the instant before the shot indicated that, in goals and saves, the goalkeeper optimized his position before the shot in 21.87% and 83.33% of the situations, respectively.

The present approach increased resolution regarding the individual action of a player, in extension to previous works that focused in forecasting the outcome probabilities of a certain game configuration both in team sports in general [14] and in soccer [20, 21]. Additionally, the use of a probabilistic framework to evaluate the decision made by the player before he performs the action may be complementary to studies that investigate the functional interpersonal interactions between attackers and defenders, based on variations in the spatial positioning of team players and adversaries relative to each other in critical performance areas [5,6]. It may be elucidating to define patterns of interaction between players, rank them based on the probability of obtaining a successful outcome and then analyze features of the interpersonal interaction between them.

Particularly in soccer, the proposed analysis complements previous studies that analyzed the goalkeeper’s actions in specific game situations [22–25]. Geometrical parameters for the goalkeeper positioning, the ball line and the goal centerline were defined. Thus, the player’s decision model (PD) used these action parameters to assess a goalkeeper’s decision in response to the offensive situation, supporting the analysis of his variability ratio. Additionally, PD supported the identification of the most successful goalkeeper’s actions (e.g., in a central-far offense, the greatest probability of a goalkeeper’s defense (0.50) was achieved in a retrieved and centralized position). The actions from PD with greatest probability of occurrence determined the player strategy, defined by the player strategy model (PS). Evaluation of the success rate of all possible actions, given a game situation, may contribute to improve the strategy of a certain player.

The success ratio of a player’s actions was assessed through the oracle. In this approach, the stochastic model PD was estimated from two different data sets: i) actions from several players (the oracle) with similar role and competitive level to the player of interest; and ii) actions from the player of interest. The smaller sample of actions from the analyzed player was evaluated according to the larger sample of success probability from the oracle. Thus, the oracle supported the estimation of the player’s decision performance (PD) and of his respective strategy performance (PS) in each situation (*x*_*i*_, *a*_*i*_). In the case of soccer goalkeepers, the estimation of the goal probability, based on the oracle, has been denominated as the goal potential. Thus, the goal potential is a reference for quantitatively comparing the performance (*x*_*i*_, *a*_*i*_) considering the average performance of the respective situation (*x*_*i*_).

The application of this analytic method required that the events be modeled as a categorical system [8]. Each category was defined by a set of parameters, which are sufficient for describing the semantics of the situation. In the specific case of the soccer goalkeeper’s performance, categories were systematized in a goalkeeper positioning model based on geometric parameters (see Fig 2). The aim of the positioning model is to shorten the distance between the goalkeeper and the ball trajectory. The categorical variables used to characterize the main intervenient elements of the decision performance were as follows: i) *x*_*i*_: offensive situation and ii) *a*_*i*_: goalkeeper action. These variables presented reliable criteria, according to the Kappa values for both intra- and inter-rater, varying between 0.88 to 1 and 0.80 to 0.95, respectively.

For the offensive situation (*x*_*i*_), the main tendencies in the oracle indicated that the three greatest probabilities for a goal occurred in the central near zone after the following situations: i) unbalanced defense—0.91; ii) cross—0.85; and iii) not cross—0.82. On the opposite end, the lowest goal probability occurred in the side far zone—0.40, with balanced defense. Other zones that were neither central near nor side far presented goal probabilities between 0.59 and 0.71. These results corroborate a previous study that related the offensive area with the prevalence of goals [20].

For the goalkeeper’s action, in regards to the centralization parameter, the centralized position of the goalkeeper led to a better performance (i.e., lower goal probability) than not centralized, respectively, for i) central far (0.58 and 0.83); ii) central near cross (0.79 and 0.97); iii) central near not cross (0.60 and 1); iv) side far (0.42 and 0.88); v) side near cross (0.42 and 0.93); vi) side near not cross (0.29 and 0.83); vii) central near with unbalanced defense (0.71 and 0.98); and viii) side near with unbalanced defense (0.49 and 0.87).

Additionally, regarding the positioning parameter, when the goalkeeper was in a *retrieved position*, he had the lowest goal probability in the situations of i) far zones—central-far (0.58), side-far (0.37); and ii) with cross—central near cross (0.67), side near cross (0.49). On the other hand, for the *advanced position*, the goalkeeper had the lowest goal probability in the situations of i) near zones not cross—central near not cross (0.73), side near not cross (0.56); and ii) unbalanced defense situations (0.74). The *out of penalty area position* also had low goal probability in unbalanced defense situations (0.80). Therefore, general rules could be applied to both goalkeeper performance parameters: centralization and positioning. For centralization, a general rule for any *x*_*i*_ is that the goalkeeper should be centralized. For positioning, the ball position (far, near) and last defensive line balance/unbalance determines the goalkeeper’s optimal position (i.e., retrieved, advanced or out of penalty area), respectively: far/cross-retrieved, near-advanced, unbalanced-advanced/out of penalty area.

The subsequent step in the estimation process referred to the PD annotation and analysis. For all *x*_*i*_, the professional goalkeepers presented PD rates between the oracle’s lowest and oracle’s average goal probabilities, except for JC’s central near not cross, which had a single event. These results are evidence of good decision-making performances of the evaluated goalkeepers. However, PS presented better performance than PD for the two goalkeepers in all *x*_*i*_, evidenced by the fact that the goalkeepers’ PS performance corresponded to the oracle’s lowest value. These results indicated that the theoretical framework that led to the formulation of PD and PS enabled the computation of the optimal goalkeeper strategy based on the available oracle.

PD data supported the calculation of the goalkeepers’ entropies. The results indicated that goalkeepers could present lower variability of actions, as PS presented a greater save probability than PD. The tendency of varying more than optimal is in accordance with other studies that identified that players can perform with more clustered play selections, switching play types less frequently [11–13]. GB and JC presented a range between 0.24–0.71 of the maximum entropy. GB was more consistent in situations inside the area (0.24–0.45) and presented greater variability in the situation central far (0.71). A possible explanation for the augmentation in central far variability is a team strategy feature that may require a greater commitment of GB with the last defensive line coverture actions, implying more displacements demands. JC was consistent in almost all situations (0.38–0.53), except by the side near not cross (0.68). JC’s side near not cross greater variability may be a consequence of the uncertainty inherent to the situation, given the goal proximity and risk of a cross instead of a shoot. Entropy results highlight the relevance of associating this variability measure to the goal potential information, as the former can beneficiate from a player consistently increase the frequency of making the appropriate decision. Results obtained with the two top professional goalkeepers analyzed evidence that even in the highest level, players can improve their decision performance consistency.

The evaluation of the GPM demonstrated a perfect agreement with the oracle’s lowest values for all evaluated *x*_*i*_, except for the *x*_*i*_ with cross situations, which were not considered in the GPM. This great performance achieved by the strategy model evidenced its potential for supporting the design of goalkeepers’ strategies.

Finally, a dynamic analysis of the events leading to a shot in goal and saves was performed, with the aim of assessing performance in the sequence of decisions, along with the states that precede the finalization. According to the oracle data, as expected, there were high upward and high downward trends of goal probability, respectively, in goal and save situations. In goal situations, 57.8% of sequences were composed by all states with a goalkeeper positioning with more than 0.50 probability of goal, according to the oracle data. Thus, in goal situations, the greatest part of the sample presented non-optimal positioning during all the sequence. Complementarily, in save situations, only 28.4% of sequences presented one or more states with a goalkeeper positioning with goal probability greater than 0.50. Thus, in save situations, although positioning errors may frequently occur, it is more likely that the goalkeeper recovers a better positioning in a following state and it positively impacts his performance.

Complementarily, two specific cases were analyzed: i) cross with finalization in central near and ii) last defensive line balanced/unbalanced. In crosses, the results indicated that if the goalkeeper centralizes before the finalization state, as the play (cross) is a fast play, he could not sustain the centralized positioning to the finalization state because the ball overcame him in all instances. In all saves, the goalkeeper was not centralized in the -first state, and in 83.4% of the instances the goalkeeper ended centralized. Additionally, in most of the goal situations, 81.3%, and in all save situations, the positioning of the goalkeeper was retrieved. A practical recommendation is that goalkeepers should leave the goal for intercepting the cross only in situations evaluated with high probability of success. In the case of balanced/unbalanced last defensive line, in goal situations the goalkeeper was centralized only in 10% of instances, and in defense situations the goalkeeper was centralized in 60%. Moreover, the results showed a strong tendency of goalkeepers to keep an advanced position in the finalization state (81% of the cases in the entire sample). Thus, the difference between success and failure in this specific situation seems to be more related to centralization.

## Conclusion

The findings of this work constitute a solution for the challenge of *a priori* evaluation of an individual player’s performance by applying a join probability distribution measure that relates states’ transitions (i.e., player action) and its success in a certain context. Theoretically, it may contribute to enhancing the accuracy of experimental designs for individual players’ decisions evaluation by considering the probability of each outcome, given the action performed by the player. The experimental results indicated that the professional goalkeepers analyzed were able to decide on positioning similarly to the best oracle results. It evidences that an efficient set of positioning rules can be defined and learned. The GPM performance suggests it can be used as an effective reference for teaching goalkeepers’ positioning rules that maximize their saves probability. Interestingly, entropy results indicated that although the goalkeepers presented a high performance in their positioning choices, they varied more frequently than optimal. This reflects the relevance of approaching the decision variability issue in the training process. This fact is corroborated by the dynamic analysis according to which non-optimal positioning instants prior to the shot increases the probability of a goal. In practice, performance analysts may benefit from this framework to set up analysis at the player level. Soccer coaches may benefit from the positioning model and analytical structure presented to predict their own goalkeepers’ success probability and increase their decision-making ability.

## Supporting information

S1 File Oracle data and professional goalkeepers data. (XLSX)
